# Supplementary material for: Joint Tissues: Convergence and Divergence of the Pathogenetic Mechanisms of Rheumatoid Arthritis and Osteoarthritis
Source: Int J Mol Sci. 2025 Sep 8;26(17):8742. doi: 10.3390/ijms26178742 (PMC12429403; doi:10.3390/ijms26178742)
Supplement: Supplementary file 1 [file ijms-26-08742-s001.zip › ijms-3814443-supplementary.docx]

**Supplementary Table S1.** Shared RA and OA associated mutated genes*

| Gene | NCBI gene information | Gene SNP and/or role in pathogenesis | | |
| --- | --- | --- | --- | --- |
|  |  | OA | | RA |
| ANAPC4 | Anaphase promoting complex subunit 4  A large protein complex, termed the anaphase-promoting complex (APC), or the cyclosome, promotes metaphase-anaphase transition by ubiquitinating its specific substrates such as mitotic cyclins and anaphase inhibitor, which are subsequently degraded by the 26S proteasome. Biochemical studies have shown that the vertebrate APC contains eight subunits. The composition of the APC is highly conserved in organisms from yeast to humans. | | Risk of OA | Risk of RA |
| ANXA3  RANKL/RANK HIF | Annexin A3  This gene encodes a member of the annexin family. Members of this calcium-dependent phospholipid-binding protein family play a role in the regulation of cellular growth and in signal transduction pathways. This protein functions in the inhibition of phospholipase A2 and cleavage of inositol 1,2-cyclic phosphate to form inositol 1-phosphate. | | Risk of OA (self-reported) | Risk of RA |
| CCDC26 | CCDC26 long non-coding RNA | | Risk of OA | Risk of RA. |
| TSBP1 (TSBP, C6orf10) | Testis expressed basic protein 1  It is expressed in the nervous system and sensory organs. | | Risk of knee OA | Risk of RA |
| BTNL2 | Butyrophilin like 2  This gene encodes a major histocompatibility complex, class II associated, type I transmembrane protein which belongs to the butyrophilin-like B7 family of immunoregulators. It is thought to be involved in immune surveillance, serving as a negative T-cell regulator by decreasing T-cell proliferation and cytokine release. The encoded protein contains an N-terminal signal peptide, two pairs of immunoglobulin-like domains, separated by a heptad peptide sequence, and a C-terminal transmembrane domain. Naturally occurring mutations in this gene are associated with sarcoidosis, rheumatoid arthritis, ulcerative colitis, inflammatory bowel disease, myositis, type 1 diabetes, systemic lupus erythematosus, acute coronary syndrome, and prostate cancer. | | Risk of knee OA | Risk of RA |
| PLCL2 | Phospholipase C like 2  Enables GABA receptor binding activity. Predicted to be involved in the gamma-aminobutyric acid signaling pathway; negative regulation of cold-induced thermogenesis; and regulation of GABAergic synaptic transmission. Predicted to act upstream of or within B cell proliferation involved in immune response; B-1a B cell differentiation; and negative regulation of B cell receptor signaling pathway. Predicted to be located in the cytoplasm. | | Risk of surgical hip OA | Risk of ACCP and/or RF positive RA |
| CCR3 | CCR3 – C-C motif chemokine receptor 3  The protein encoded by this gene is a receptor for C-C type chemokines. It belongs to family 1 of the G protein-coupled receptors. This receptor binds and responds to a variety of chemokines, including eotaxin (CCL11), eotaxin-3 (CCL26), MCP-3 (CCL7), MCP-4 (CCL13), and RANTES (CCL5). It is highly expressed in eosinophils and basophils, and is also detected in TH1 and TH2 cells, as well as in airway epithelial cells. This receptor may contribute to the accumulation and activation of eosinophils and other inflammatory cells in the allergic airway. It is also known to be an entry co-receptor for HIV-1. This gene and seven other chemokine receptor genes form a chemokine receptor gene cluster on the chromosomal region 3p21. | | Risk of OA | Risk of severe RA |
| ABHD16B | Abhydrolase domain containing 16B  Predicted to enable monoacylglycerol lipase activity and phospholipase activity. Predicted to be involved in the monoacylglycerol catabolic process and phosphatidylserine catabolic process. Located in the nucleoplasm. | | Risk of knee OA, risk of obesity | Risk of aCCP/RF positive RA |
| CSMD1 | CUB and Sushi multiple domains 1  Predicted to act upstream of or within several processes, including learning or memory; mammary gland branching involved in pregnancy; and reproductive structure development. Predicted to be located in membrane. | | Risk of RA OA, high levels of serum cartilage oligomeric protein and urinary C-telopeptide of type II collagen | Risk of joint destruction in RA (rapid vs slow) |
| BLK | BLK proto-oncogene, Src family tyrosine kinase  This gene encodes a nonreceptor tyrosine-kinase of the src family of proto-oncogenes that are typically involved in cell proliferation and differentiation. The protein has a role in B-cell receptor signaling and B-cell development. The protein also stimulates insulin synthesis and secretion in response to glucose and enhances the expression of several pancreatic beta-cell transcription factors | | Risk of OA | Risk of RF and/or ACCP seropositive RA |
| JAZF1 | JAZF zinc finger 1  This gene encodes a nuclear protein with three C2H2-type zinc fingers, and functions as a transcriptional repressor. Chromosomal aberrations involving this gene are associated with endometrial stromal tumors. | | Risk of hip OA and obesity | Risk of RA |
| DLG2 | Discs large MAGUK scaffold protein 2  This gene encodes a member of the membrane-associated guanylate kinase (MAGUK) family. The encoded protein forms a heterodimer with a related family member that may interact at postsynaptic sites to form a multimeric scaffold for the clustering of receptors, ion channels, and associated signaling proteins. | | Risk of OA | Associated with response to anti-TNF therapy (change in swollen 28-joint count) |
| COL11A2 | Collagen type XI alpha 2 chain  This gene encodes one of the two alpha chains of type XI collagen, a minor fibrillar collagen. It is located on chromosome 6 very close to but separate from the gene for retinoid X receptor beta. Type XI collagen is a heterotrimer but the third alpha chain is a post-translationally modified alpha 1 type II chain. Proteolytic processing of this type XI chain produces PARP, a proline/arginine-rich protein that has an amino terminal domain. | | Risk of OA | Risk of RA, risk of diabetes |
| FTO | FTO alpha-ketoglutarate dependent dioxygenase  This gene is a nuclear protein of the AlkB related non-heme iron and 2-oxoglutarate-dependent oxygenase superfamily but the exact physiological function of this gene is not known. Other non-heme iron enzymes function to reverse alkylated DNA and RNA damage by oxidative demethylation. Studies in mice and humans indicate a role in nervous and cardiovascular systems and a strong association with body mass index, obesity risk, and type 2 diabetes | | Risk of hip OA, risk of obesity. | Associated with response to anti-TNF therapy (change in swollen 28-joint count) |
| TYK2 | Tyrosine kinase 2  This gene encodes a member of the tyrosine kinase and, more specifically, the Janus kinases (JAKs) protein families. This protein associates with the cytoplasmic domain of type I and type II cytokine receptors and promulgates cytokine signals by phosphorylating receptor subunits. It is also a component of both the type I and type III interferon signaling pathways. As such, it may play a role in anti-viral immunity. | | Risk of OA | Associated with response to anti-TNF therapy, joint damage |
| CCR2 | C-C motif chemokine receptor 2  The protein encoded by this gene is a receptor for monocyte chemoattractant protein-1, a chemokine which specifically mediates monocyte chemotaxis. Monocyte chemoattractant protein-1 is involved in monocyte infiltration in inflammatory diseases such as rheumatoid arthritis as well as in the inflammatory response against tumors. The encoded protein mediates agonist-dependent calcium mobilization and inhibition of adenylyl cyclase. | | Risk of OA | Associated with response to anti-TNF therapy (change in swollen 28-joint count) |
| HLA-DPB1 | Major histocompatibility complex, class II, DP beta 1  HLA-DPB belongs to the HLA class II beta chain paralogues. This class II molecule is a heterodimer consisting of an alpha (DPA) and a beta chain (DPB), both anchored in the membrane. It plays a central role in the immune system by presenting peptides derived from extracellular proteins. Class II molecules are expressed in antigen presenting cells (APC: B lymphocytes, dendritic cells, macrophages). | | Risk of knee OA | Risk of arthritis. |
| GLIS3 | GLIS family zinc finger 3  This gene is a member of the GLI-similar zinc finger protein family and encodes a nuclear protein with five C2H2-type zinc finger domains. This protein functions as both a repressor and activator of transcription and is specifically involved in the development of pancreatic beta cells, the thyroid, eye, liver and kidney. Mutations in this gene have been associated with neonatal diabetes and congenital hypothyroidism (NDH). | | Associated with predisposition to post-traumatic knee OA development. | Risk of RA. |
| TGFA | Transforming growth factor alpha  This gene encodes a growth factor that is a ligand for the epidermal growth factor receptor, which activates a signaling pathway for cell proliferation, differentiation and development. This protein may act as either a transmembrane-bound ligand or a soluble ligand. This gene has been associated with many types of cancers. | | Risk of hip/knee OA | Risk of severe RA progression |
| LINC01016 | Long intergenic non-protein coding RNA 1016 | | Risk of idiopathic knee OA | Risk of RA |
| PTPRM | protein tyrosine phosphatase receptor type M  The protein encoded by this gene is a member of the protein tyrosine phosphatase (PTP) family. PTPs are known to be signaling molecules that regulate a variety of cellular processes including cell growth, differentiation, mitotic cycle, and oncogenic transformation. This PTP possesses an extracellular region, a single transmembrane region, and two tandem catalytic domains, and thus represents a receptor-type PTP. The extracellular region contains a meprin-A5 antigen-PTP mu (MAM) domain, an Ig-like domain and four fibronectin type III-like repeats. This PTP has been shown to mediate cell-cell aggregation through the interaction with another molecule of this PTP on an adjacent cell. This PTP can interact with scaffolding protein RACK1/GNB2L1, which may be necessary for the downstream signaling in response to cell-cell adhesion. | | Risk of OA, risk of obesity | Associated with response to methotrexate |
| DLGAP2 | DLG associated protein 2  The product of this gene is a membrane-associated protein that may play a role in synapse organization and signaling in neuronal cells. | | Risk of self-reported OA | Risk of RA |
| SENP1 | SUMO specific peptidase 1  This gene encodes a cysteine protease that specifically targets members of the small ubiquitin-like modifier (SUMO) protein family. This protease regulates SUMO pathways by deconjugating sumoylated proteins. This protease also functions to process the precursor SUMO proteins into their mature form. | | Risk of knee OA with total replacement | Risk of RA |
| COL2A1 | collagen type II alpha 1 chain  This gene encodes the alpha-1 chain of type II collagen, a fibrillar collagen found in cartilage and the vitreous humor of the eye. Mutations in this gene are associated with achondrogenesis, chondrodysplasia, early onset familial osteoarthritis, SED congenita, Langer-Saldino achondrogenesis, Kniest dysplasia, Stickler syndrome type I, and spondyloepimetaphyseal dysplasia Strudwick type. In addition, defects in processing chondrocalcin, a calcium binding protein that is the C-propeptide of this collagen molecule, are also associated with chondrodysplasia. | | Risk of knee OA with total replacement | Risk of RA |
| ITPR3 | inositol 1,4,5-trisphosphate receptor type 3  This gene encodes a receptor for inositol 1,4,5-trisphosphate, a second messenger that mediates the release of intracellular calcium. The receptor contains a calcium channel at the C-terminus and the ligand-binding site at the N-terminus. Knockout studies in mice suggest that type 2 and type 3 inositol 1,4,5-trisphosphate receptors play a key role in exocrine secretion underlying energy metabolism and growth | | Risk of idiopathic knee OA. | Risk of RA, risk of diabetes melitus |
| LINC02341 | long intergenic non-protein coding RNA 2341 | | Risk of knee OA | Risk of RA |
| IL4R | IL4 receptor | | Risk of hip OA | Risk of RA |
| IL-4 | Anti-inflammatory cytokine | | Risk of knee OA, risk of hand OA | Risk of RA  Risk of early joint destruction |
| SMAD3 | SMAD family member 3  SMAD family of proteins are a group of intracellular signal transducer proteins. The SMAD3 protein functions in the transforming growth factor-beta signaling pathway and transmits signals from the cell surface to the nucleus, regulating gene activity and cell proliferation. This protein forms a complex with other SMAD proteins and binds DNA, functioning both as a transcription factor and tumor suppressor. Mutations in this gene are associated with aneurysms-osteoarthritis syndrome intracellular mediator regulating extracellular TGF-β signals to transduce into nucleus. TGF-β/SMAD3 signaling is crucial to maintain the integrity of articular cartilage | | Risk of knee OA | Associated with RA susceptibility, activity, joint damage and extra-articular manifestation. |

*The information got in GWAS and NCBI

**Supplementary Table S2.** Key words for the search of RA and OA link with non-genetic risk factors in PubMed

| Disease | Non-genetic trigger | Statistical indicator |
| --- | --- | --- |
| RA ACCP-positive | Family aggregation | OR/HR/RR |
|  | Perinatal and/or early life factors: parents smoking during pregnancy, birth weight |  |
|  | Age |  |
|  | Gender |  |
| RA ACCP-negative | Various gender associated factors: childbirth, menopause, oral contraceptive pills, postmenopausal hormone therapy |  |
| Osteoarthritis | Body mass index (BMI) |  |
|  | Alcohol |  |
|  | Smoking |  |
|  | Mental stress |  |
|  | Physical activity |  |

**Supplementary Table S3.** RA and OA links with non-genetic risk factors

|  | ACCP-positive RA | ACCP-negative RA | | | OA |
| --- | --- | --- | --- | --- | --- |
| Family Aggregation | Familial risk OR=**3.7*** (95% CI 2.9-4.7), n=1,652  <40 years OR=**6.2*** (95% CI 3.5-10.9)  40-60 years OR=**3.3*** (95% CI 2.4-4.6)  60+ years OR=**3.3** *(95% CI 2.0-5.3) [109] | Familial risk OR=**2.1*** (95% CI 1.5-3.1), n=873  <40 years OR=**3.5*** (95% CI 1.4-9.0)  40-60 years OR=**2.1*** (95% CI 1.2-3.6)  60+ years OR=1.8 (95% CI 1.0-3.3) [109] | | | Familial risk OR = **4.4*** (95% CI 2.0-9.5), hip OR=3.9* (95% CI 1.8-8.4), spine OR = 2.2 (95% CI 1.0-5.1) adjusted for age, sex, and body mass index [110]; knee OR= **3.61** (95% CI 2,69-4.85) [111] |
| Age | Age (50-59, vs 30-39) HR=**2.445*** (95% CI 1.909-3.131) [115] | Z | | | Z |
|  | vs 25-44 years  45-49 RR=1.2 (95% CI 0.9-1.7)  50-54 RR=2.0* (95% CI 1.5-2.7)  55-59 RR=2.1* (95% CI 1.5-2.8)  60-64 RR=1.4 (95% CI 0.6-3.2) [112] | vs 25-44 years  45-49 RR=2.1* (95% CI 1.5-3.0)  50-54 RR=2.0* (95% CI 1.3-3.0)  55-59 RR=2.7* (95% CI 1.4-5.4)  60-64 RR=2.5* (95% CI 1.5-4.2)[112] | | | Prevalence of OA (%)  40 to < 45 13.2% (95% CI 10-16)  45 to < 50 16.5% (95% CI 14-19)  50 to < 55 21.8% (95% CI 19-24)  55 to < 60 27.4% (95% CI 25-30)  60 to < 65 32.9% (95% CI 30-36)  65 to < 70 36.2% (95% CI 33-40)  75 to < 80 38.8% (95% CI 34-44) [113] |
| Gender Associated Factors | Male vs female HR=0.387* (95% CI 0.340-0.441) [115] | Z | | | Male vs female OR=2.87 (95% CI 1.94-4.25) [111]  Incidence rate of knee OA IRR=0.55* (95% CI 0.32-0.94), hip OA IRR=0.64* (95% CI 0.48-0.86), and a **non-significant** reduction hand OA IRR=0.65 (95% CI 0.35-1.20)[116] |
|  | Gender-specific prevalence: **1.54***% (95% CI, 1.4-1.69) for females; **0.4***% (95% CI, 0.32-0.49) for males [114] | | | | Age-standardized prevalence of hand OA modestly higher in women (44.2%) vs men (37.7%), age-standardized prevalence of erosive and symptomatic OA higher in women **(9.9*% vs 3.3%, and 15.9*% vs 8.2%)** [152] |
|  | Postmenopausal vs premenopausal women HR=1.2 (95% CI 0.9-1.6) [112] | | | Postmenopausal vs premenopausal women HR=2.1* (95% CI 1.4-3.0) [112] | Spontaneous/surgical menopause vs premenopausal OR=**1.13*** (95% CI 1.07-1.21)/ OR=1.18* (95% CI 1.08-1.28) [118] |
|  | Age at menopause vs pre-menopausal  ≤44 years HR=1.2 (95% CI 0.9-1.7)  45-49 years HR=1.2 (95% CI 0.9-1.7)  ≥ 50 years HR=1.2 (95% CI 0.9-1.7) [112] | | | Age at menopause vs pre-menopause)  ≤44 years HR=2.1* (95% CI 1.4-3.1)  45-49 years HR=1.7* (95% CI 1.1-2.7)  ≥ 50 years HR=2.0* (95% CI 1.2-3.1) [112] | Z |
|  | Postmenopausal hormones  current users vs never users HR= 1.3 (95% CI 0.9-1.8) no effect [112] | | | Postmenopausal hormones  current users vs never users HR= 1.3 (95% CI 0.9-1.8) no effect [112] | Postmenopausal hormones  current users vs never users OR=**0.70*** (95% CI 0.50-0.99) [121] |
|  | **Estrogen plus progestogens** therapy users vs never users OR=**0.3*** (95% CI 0.1-0.7) [120] | | | **Estrogen plus progestogens** therapy users vs never users OR=1.0 (95% CI 0.7-1.4) [120] | **Estrogen plus progestogens** users vs non users HR=1.092 (95% CI, 1.048-1.137) [123] |
|  |  |  |  |  | **Estrogens** therapy users vs never users OR=**0.73*** (95% CI 0.75-0.84)[118] ; HR=**1.235*** (95% CI, 1.148-1.329) [123];  risk of knee OA RR=**1.8*** (95% CI 1.2-2.6) [58]; risk of hip OA OR =5.03, (95% CI 1.70-14.84) hand OA OR=**1.57** (95% CI 1.05-2.33) [124] |
|  | Parous vs nulliparous RR=0.96 (95% CI 0.89-1.04) [125] | | | | Parous vs nulliparous R=**1.031***(95% CI 1.023-1.039) [129] |
|  | Parous vs nulliparous women aged 18-44 years R=0.9 (95% CI 0.7-1.2) [126] | | | Parous vs nulliparous women aged 18-44 years OR=**2.1*** (95% CI 1.4-3.2) [126] |  |
|  | 2 children vs 1 RR=**0.84*** (95% CI 0.78-0.90)  3 children vs 1 RR=**0.83*** (95% CI 0.77- 0.91) [125] | | | | 1-2 children vs none R=**1.49*** (95% CI 1.22-1.82)  3-4 children vs none R=**3.26*** (95% CI 2.68-3.96) [128] |
|  | vs normal pregnancy history  **Pregnancies complicated** by hyperemesis RR=1.70* (95% CI 1.06-2.54), gestational hypertension RR=**1.5*** (95% CI 1.06-2.02), pre-eclampsia RR=1.42* (95% CI 1.08-1.84).  Risk of RA increased significantly with increasing number of pregnancies complicated by gestational hypertension or pre-eclampsia (p for trend = 0.003) [125] | | | | Z |
|  | **Breast feeding** (yes/no) OR=0.2* (95% CI 0.1-0.7) [133] | | | | **Breast feeding** (yes/no) OR=**1.55*** (95% CI 1.18-2.03)[128];  risk of knee OA OR=**2.30*** (95% CI 1.09-4.86)[134] |
|  | **Breast feeding** for 1-12 months vs 0 months OR=0.74* (95% CI 0.41-1.35) [199]; HR=**0.66*** (95% CI 0.46- 0.94) [142] | | | | Z |
|  | **Breast feeding** for 13 months or more vs 0-6 months OR=**0.77** (95% CI 0.63-0.94) [131] | | | **Breast feeding** for 13 months or more vs 0-6 months OR=0.91 (95% CI 0.65-1.27) [131] | Z |
|  | Breast feeding for >13 months vs 0 months **0.47*** (95% CI 0.19-1.14) [304] | | | |  |
|  | **Breast feeding** for 24 months vs 0 months OR= **0.5** (95% CI 0.3-0.7) [132] | | | |  |
|  | **Oral contraceptives** users vs never users OR=**1.65*** (95% CI 1.06-2.57) [130] | | | **Oral contraceptive** users vs never users OR=1.19 (95% CI 0.68-2.07) [130] | **Oral contraceptives** users vs never users OR=0.9 (95% CI 0.6-1.4)[122] ; OR=0.972 (95% CI 0.967-0.977) [129] |
|  | **Oral contraceptives** ever vs never users OR=**0.84** (95% CI 0.74-0.96)[131] | | | **Oral contraceptives** ever vs never users OR=0.93 (95% CI 0.79-1.10) [131] |  |
|  | RA risk ever, current and past **Oral contraceptives** users vs never users OR=1.00 (95% CI 0.87-1.15), OR=0.93 (95% CI 0.70-1.23) and OR=0.93 (95% CI 0.78-1.12), respectively [305] | | | | Z |
| Perinatal Factors | **Birth weight**  **>** 4.54 kg (vs 3.2-3.85 kg) RR=2.1 (95% CI 1.4-3.3)[137]  > 4 kg (vs 3-3,9) OR=3.6* (95% CI 1.4-9.1)  Large for gestational age (yes/no) OR=4.4* (95% CI 1.6-12)  Small for gestational age (yes/no) OR=1.0 (95% CI 0.3-2.6) [133] | | | | **Birth weight**  **Lower birth** weight and osteophytes in hip OR=1.51* (95% CI 1.13-2.01)  clinical hand OA OR=1.396* (95% CI 1.05-1.85) [140]; HR=2.02 (95% CI 1.10-3.73) [139]  Preterm birth HR=2.53* (95% CI 1.30-4.92) [139] |
| Body Mass Index | BMI ≥25 <30 kg/m2 OR=0.8 (95% CI 0.7-1.0), women OR=1.0 (95% CI 0.8-1.2) [141]; OR=1.01 (95% CI 0.69-1.47) [130] | | BMI ≥25 <30 kg/m2 OR=**1.4*** (95% CI 1.1-1.9), women OR=**1.6*** (95% CI 1.2-2.2) [141]; HR **2.75*** (95% CI 1.39-5.46) for obese vs normal-weight [142]; OR=1.24 (95% CI 0.74-2.10) [130] | | BMI ≥25 <30 kg/m2 hip OA RR=1.04 (95% CI 1.00-1.07) and joint surgery RR=1.16 (95% CI 1.11-1.22) not reliable [143];  >25 kg/m2 OR=3.29* (95% CI 2.40-4.51) [111]; knee OA OR=2.68* (95% CI 2.33-3.09); hip OA OR=1,65* (95% CI 1.46-1.87) [145] |
|  | ≥30 kg/m2 OR=1.15 (0.62 to 2.13) [130]; 30-34.9 kg/m2 HR=**0.583*** (95% CI 0.440-0.772) [115] | | ≥30 kg/m2 OR=**3.45*** (95% CI 1.73-6.87) [130] | | > 30 kg/m2 knee OA OR=**2.81*** (95% CI 1.32-5.96), hip OA OR=1.11 (95% CI 0.41-2.97), hand OA OR=**2.59*** (95% CI 1.08-6.19)[146] ; knee OA OR=7.48* (95% CI 5.45-10.27) [145] |
|  | Z | | Z | | Risk associated with a 5 kg/m2 increase in BMI: hand OR=**1.25*** (95% CI 1.06-1.49) [147] ; knee OA RR=**1.25*** (95% CI 1.17-1.35) [144] |
| Coffee consumption | Dose dependent effect*  0-5 cups/day 1.20 (95% CI 0.76-1.91)  5-10 cups/day **1.70** (95% CI 0.95-3.05) >10 cups/day **2.18** (95% CI 1.07-4.42) [130] | | Dose dependent effect  0-5 cups/day 0.79 (95% CI 0.41-1.52)  5-10 cups/day 0.94 (95% CI 0.47-1.90)  >10 cups/day 1.23 (95% CI 0.48-3.16) [130] | | 1 cup/day knee OA OR=**1.023*** (95% CI 1.009-1.038) [148]; OR=1.01 (95% CI 0.999-1.025) [149]  Men: < 2 cup/day OR=1.13 (95% CI 0.50-2.55), 2-3 cup/day OR=1.79 (95% CI 0.81-3.97), 4-6 cup/day OR=2.21 (95% CI 0.91- 5.35), and ≥ 7 cup/day OR=**3.81** (95% CI 1.46-12.45) .  Women - no association with studied doses [149] |
| Alcohol | Drinkers vs non-drinkers OR=**0.52*** (95% CI 0.36-0.76) [306] | | Drinkers vs non-drinkers OR= 0.74 (95% CI 0.53-1.05) not reliable [306] | | Z |
|  | Unit/day vs non-drinker HR=**0.86*** (95% CI 0.74-0.99) [142] | | | | Z |
|  | 5-10 cups/week vs non-drinker HR=**0.810*** (95% CI 0.657-0.999) [115] | | | Z |  |
|  | ≥4 doses/ week vs non-drinkers OR=**0.64*** (95% CI 0.36-1.14) [150] | | | Z |  |
|  | 0 drinks/week vs 0-5 drinks/week OR=**1.18*** (95% CI 0.82-1.71)  > 15 drinks/week vs > 0-5 drinks/week  OR=**0.66*** (95% CI 0.24-1.84) [130] | | | 0 drinks/week vs > 0-5 drinks/week OR=0.98 (95% CI 0.62-1.55)  >15 drinks per week vs >0 to 5 drinks per week **OR=1.36*** (95% CI 0.54-4.66) [130] | 1-6 drinks/week vs 0 drinks/week hand OA OR= **1.82*** (95% CI 0.99-3.36) [151]  1-3 drinks/week vs 0 drinks/week hand OA OR= **1.55*** (95% CI 0.43-2.67) [152] |
| Smoking | Ever smokers vs never smokers:  RR=**1.5*** (95% CI 0.8-2.9) - **21*** (95% CI 11.0-40.2) (no SE-double SE) [153];  OR=**4.1*** (95% CI 1.9-9.2) [154] | | | Ever smokers vs never smokers:  RR= **0.6*** (95% CI 0.4-1.0) - 0.8 (95% CI 0.4-1.7) (no SE-double SE) [153];  OR=0.7 (95% CI 0.3-2.0) [154] | Ever smokers vs never smokers knee OA  RR=**0.80*** (95% CI 0.73-0.88) [156] |
|  | Current smoking vs nonsmokers  OR=**2.13*** (95% CI 1.54-2.95) [130] | | | Current smoking vs nonsmokers OR=1.01 (95% CI 0.65-1.57) [130] | Z |
|  | >10 to 20 pack-years OR=**2.41*** (95% CI 1.51-3.82) [130]  1-19 pack-years OR=**3.3*** (95% CI 1.1-9.8),  ≥20 pack-years OR=**5.5*** (95% CI 1.6-17.6) [154] | | | >10 to 20 pack-years OR=**0.72***(95% CI 0.37-1.40) [130]  1-19 pack-years OR=1.9 (95% CI 0.5-8.0),  ≥20 pack-years OR=0.4 (95% CI 0.1-2.3) [154] | 1-10/day knee OA OR=0.91 (95% CI 0.68-1.21) [155]  10-20/day knee OA OR=**0.566*** (95% CI 0.470-0.683) [157]  11-20/day knee OA OR=**0.70*** (95% CI 0.52-0.94) [155] |
| Mental stress | Depression prior RA onset (yes/no) HR=**1.38*** (95% CI 1.31-1.46), antidepressant use before RA onset (yes/no) HR=0.74* (95% CI 0.71-0.76) [159]  exposure during 5-year period preceding the diagnosis of RA vs no OR=**8.88*** (95% CI 1.6-47.6) [158] | | | | Z |
|  | Female  Depression prior RA onset (yes/no)  HR=1.12 (95% CI 0.93-1.35) [160] | | | Female  Depression prior RA onset (yes/no)  HR=**1.63***(95% CI 1.27-2.09) [160] | Female  Depressive mood (yes/no)  OR=**2.80*** (95% CI 1.31-3.31),  Psychological distress (yes/no)  OR=**1.92*** (95% CI 1.21-3.05)  Male  Depressive mood (yes/no)  OR=**1.51*** (95% CI 1.16-1.95)  Psychological distress (yes/no)  OR=**1.36*** (95% CI 1.07-1.72) [162] |
|  |  |  |  |  | Knee/hip  high stress perception  male OR=**1.59***(95% CI 1.10-2.31)  female OR=**1.41*** (95% CI 1.19-1.68)  depression  male OR=**1.52*** (95% CI 1.01-2.29)  female OR=**1.27*** (95% CI 1.04-1.54) [161] |
| Physical activity | Physical activity at work (ten years prior to interview)  Slight - OR=1.10 (95% CI 0.65-1.87)  High - OR=1.18 (95% CI 0.68-2.06) [130] | | | Physical activity at work (ten years prior to interview)  Slight - OR=**1.41*** (95% CI 0.74-2.67)  High - OR=**1.33*** (95% CI 0.66-2.67) [130] | Stair climbing >10 flights/d OR=**6.08*** (95% CI 4.16-8.89) [111]  Walking >2 miles/day OR=**1.9*** (95% CI 1.4-2.8) [164]  Occupational lifting and hip OA  regular lifting of  25 kg OR=**3.6*** (95% CI 1.3-9.7)[163] ; OR=**1.7*** (95% CI 1.2-2.6) [164];  50 kg in main job OR = **4.0*** (95% CI 1.1-14.2) [163] |
